# Classic Psychedelics for Treating Chronic Pain: Mechanism and Clinical Translation

**DOI:** 10.3390/molecules31152611

**Published:** 2026-07-27

**Authors:** Hongyu Chen, Bowen Ke, Ruotian Jiang

**Affiliations:** 1 Department of Anesthesiology, West China Hospital, Sichuan University, Chengdu 610000, China; hongychen@stu.scu.edu.cn; 2 Laboratory of Anesthesia and Critical Care Medicine, National-Local Joint Engineering Research Center of Translational Medicine of Anesthesiology, West China Hospital, Sichuan University, Chengdu 610000, China

**Keywords:** classic psychedelics, chronic pain, analgesia, 5-HT receptor, neuroplasticity, default mode network

## Abstract

Chronic pain is a complex disorder of central nervous system maladaptation, perpetuated not only by ascending nociceptive transmission but also by entrenched prior expectations. Conventional analgesics typically fail to reverse this cognitive rigidity and the ensuing pathological cycles. Classic psychedelics, however, are emerging as a promising avenue to help address this therapeutic impasse. This narrative review provides a comprehensive overview of the emerging science of psychedelics, encompassing their biological binding targets, potential neural mechanisms and clinical applications, in the context of chronic pain. We synthesize the therapeutic potential of classic psychedelics and critically examine the discrepancies between clinical outcomes and foundational mechanistic research. Furthermore, we evaluate key translational barriers, particularly compromised blinding and expectancy bias, and highlight the necessity for developing novel validation strategies. Ultimately, these new insights compel a fundamental rethinking of how biological neuroplasticity and subjective psychological experiences independently or synergistically drive analgesia, thereby offering a paradigm-shifting perspective for the translation of psychedelics into evidence-based analgesic therapeutics.

## 1. Introduction

According to the International Association for the Study of Pain (IASP), chronic pain is defined as pain that persists or recurs for longer than 3 months and has become recognized as a disease entity rather than merely a symptom of tissue injury [1]. The management of chronic pain remains one of the most formidable challenges in modern medicine. Chronic pain is not merely the continuation of tissue injury but rather an individual subjective experience deeply intertwined with sensory-discriminative, affective-motivational, and cognitive-evaluative dimensions [2,3,4]. Clinical evidence demonstrates that cognitive states, including attention, prior expectations and emotional contexts, such as depression and anxiety, can significantly amplify or suppress pain perception via endogenous descending modulatory systems [2,5,6]. However, the chronification of pain drives diverse, multi-scale neural remodeling, from ectopic action potentials to the formation of pathological circuits that structurally and functionally alter higher-order affective regions, such as the anterior cingulate cortex (ACC) and prefrontal cortex (PFC), thereby locking the brain into a cycle of cognitive rigidity and maladaptive prior expectations [7,8,9,10,11,12]. Current analgesics fail to address the central neural remodeling and affective disorders of chronic pain, often resulting in suboptimal efficacy and severe adverse effects [13,14,15,16]. This bottleneck compels a paradigm shift from symptomatic relief to the active remodeling of maladaptive neural networks.

Psychedelics are at the forefront of this transition. Characterized as a class of psychoactive substances capable of profoundly altering thought, perception, and emotion without inducing memory impairment, delirium, or addiction, psychedelics primarily exert their therapeutic effects by interacting with the serotonergic (5-HT, 5-hydroxytryptamine) system [17,18]. The 5-HT system comprises seven receptor families (5-HT1–5-HT7), encompassing 14 recognized receptor subtypes, several of which are established therapeutic targets in clinical practice [19]. For example, 5-HT1B/1D receptor agonists (triptans) are widely used for the acute treatment of migraine, 5-HT3 receptor antagonists are key components of antiemetic regimens for the prevention of chemotherapy-induced nausea and vomiting, while 5-HT4 receptor agonists are used to enhance gastrointestinal motility [20,21,22,23,24]. Building upon this established foundation of serotonergic pharmacology, classic psychedelics differentiate themselves by primarily targeting the 5-HT2 receptor family (most notably the 5-HT2A subtype), although interactions with other serotonergic receptor subtypes also contribute to their complex pharmacological profile [25,26]. Historically constrained by socio-historical factors and strict legal regulations, these once-marginalized 5-HT2A receptor agonists have only in recent years garnered significant attention due to their potent capacity to induce structural and functional neuroplasticity [27,28], demonstrating broad clinical potential across a variety of conditions, including psychiatric disorders, addiction, and pain [18,29,30,31,32,33,34]. The pharmacological mechanisms of psychedelics are highly complex, involving synergistic interactions across diverse neuronal populations and molecular targets within the brain, as well as significant alterations in functional connectivity between distinct brain regions. This profound neural remodeling is postulated to facilitate the breakdown of cognitive boundaries and induce ego dissolution, thereby offering substantial promise for disrupting the cycle of cognitive rigidity and entrenched prior expectations, ultimately restructuring the maladaptive neural networks characteristic of chronic pain. This narrative review comprehensively summarizes recent preclinical experiments and clinical trials investigating classic psychedelics across various types of chronic pain. Building upon the antinociceptive potential revealed by these studies, we further synthesize and postulate the distinct analgesic mechanisms by which psychedelics may operate within both the peripheral and central nervous systems. Finally, we critically discuss the unresolved challenges and ongoing controversies surrounding their clinical translation.

## 2. Classification and Receptor Profiles of Classic Psychedelics

**Figure 1:**
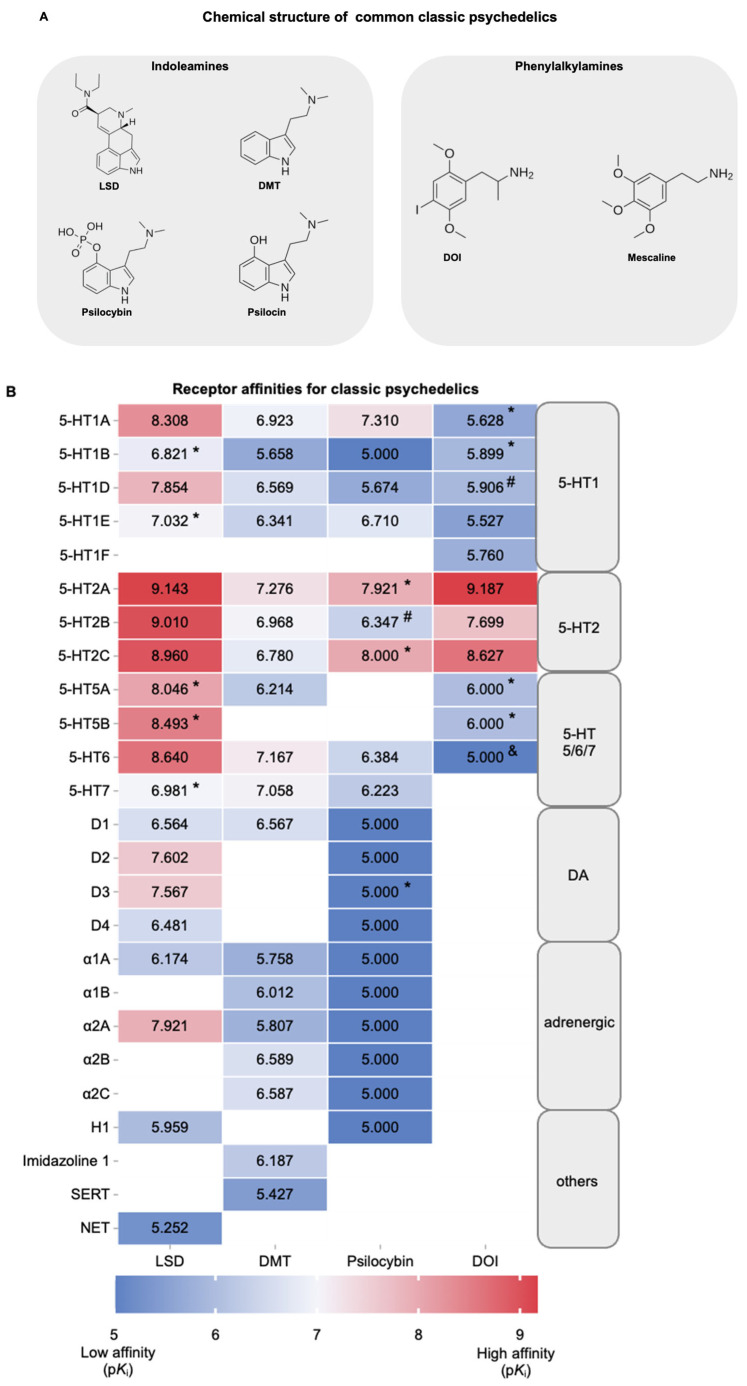
Chemical structures of common classic psychedelics and receptor affinities (all receptor affinity data presented in this figure were retrieved from the Psychoactive Drug Screening Program Ki Database (PDSP KiDB)). (**A**) Representative compounds are grouped according to their major chemical scaffolds. (**B**) This heatmap illustrates the binding affinities of four classic psychedelics (LSD, DMT, psilocybin, and DOI) across various serotonergic, dopaminergic, adrenergic, and other pharmacological targets. To effectively visualize binding data that span multiple orders of magnitude, the original inhibition constant (*K*i) values were logarithmically transformed into standard pharmacological p*K_i_* values. This logarithmic scale allows for a standardized and intuitive comparison of drug–receptor interactions. The color gradient reflects the strength of the binding affinity: warmer colors (red) denote higher p*K_i_* values, signifying stronger receptor binding affinity, whereas cooler colors (blue) indicate lower p*K_i_* values and comparatively weaker binding. A p*K_i_* value of 5 on the color scale is assigned to represent *K_i_* > 10,000 nM, indicating functionally negligible or absent binding. White cells denote that experimental binding data for that specific ligand–receptor pair are currently unavailable in the literature. Data are from human sources unless indicated otherwise: * (rat), # (bovine), and & (mouse). Abbreviations: α, alpha-adrenergic receptor; DA/D, dopamine receptor; H, histamine receptor; M, muscarinic acetylcholine receptor; SERT, serotonin transporter; NET, norepinephrine transporter. Note: Psilocybin is a prodrug that is rapidly dephosphorylated in vivo into its psychoactive and pharmacologically active metabolite, psilocin. While often used interchangeably in the literature, psilocin is the actual molecule mediating receptor binding and downstream mechanisms [42]. This figure was created and assembled by the authors using Adobe Illustrator 2023, Microsoft PowerPoint 16.89.1 and GraphPad Prism 10.

Based on the chemical structures of classic psychedelics, they are broadly classified into two major categories: indoleamines and phenylalkylamines [17,35]. Indoleamines, which primarily include lysergic acid diethylamide (LSD), N,N-dimethyltryptamine (DMT), and psilocybin, exhibit a relatively broad selectivity profile for 5-HT receptors (Figure 1A,B). They display moderate to extremely high binding affinity for 5-HT1A and 5-HT2A receptor subtypes and engage 5-HT1B/1D/1E and 5-HT2B/2C receptors. In contrast, phenylalkylamines, such as 2,5-dimethoxy-4-iodoamphetamine (DOI) and 3,4,5-trimethoxyphenethylamine (mescaline), exhibit high selectivity for the 5-HT2 receptor family (comprising the 5-HT2A, 5-HT2B, and 5-HT2C receptor subtypes) (Figure 1A). Notably, certain compounds within this class demonstrate over 1000-fold selectivity for agonist-labeled 5-HT2 receptors relative to 5-HT1 receptors [17,25,26] (Figure 1B).

Although the 5-HT2 receptor family plays an indispensable and foundational role in the pharmacological mechanisms of psychedelics, recent comprehensive G protein-coupled receptor (GPCR) screening has revealed that classic psychedelics and their derivatives possess broad affinities not only for the serotonergic system but also for a diverse array of monoaminergic receptors, including dopamine receptors (particularly D1 and D2) and adrenergic receptors (α1A and α2A) [25]. These receptor sites may collectively mediate the overall positive impact of these compounds on chronic pain. The latest single-nucleus RNA sequencing evidence further corroborates the profound interplay between psychedelics and multiple other neurotransmitter systems: In the medial prefrontal cortex (mPFC), DOI triggers not merely an isolated response in 5-HT2A receptor-positive cells but rather a widespread cascade reaction spanning multiple excitatory and inhibitory neuronal subtypes [36]. At the behavioral level, the analgesic effects of plant extracts like ayahuasca have also been shown to depend on the synergistic interaction between the γ-aminobutyric acid type A (GABAA) receptor and the serotonergic system [37]. In addition, psychedelics can directly bind to and activate tropomyosin receptor kinase B (TrkB), the receptor for brain-derived neurotrophic factor (BDNF), with affinities far exceeding those of traditional antidepressants, thereby exerting potent and rapid-acting antidepressant effects [38].

Beyond their broad multi-target binding profiles, direct physical interactions and profound signaling crosstalk exist among the diverse receptors engaged by psychedelics. A pivotal study demonstrated that serotonin 5-HT2A receptors and metabotropic glutamate 2 receptors (mGluR2) assemble via specific transmembrane domains to form functional heteromeric complexes in the brain cortex. Crucially, the activation of the mGluR2 component within this complex effectively abolishes hallucinogen-specific signaling and behavioral responses, highlighting a direct intracellular integration of serotonergic and glutamatergic pathways [39]. Furthermore, the activation of the 5-HT2A receptor induces specific phosphorylation of mGluR2 at the Ser843 residue, thereby recalibrating the signal output from an initially excitatory state towards a more inhibitory, Gi/o-mediated signaling cascade, culminating in divergent biological outcomes [40]. Recent research highlights that multiple ibogaine analogues (ibogalogs) exquisitely exploit this exact heterodimerization mechanism to effectively reverse chemotherapy-induced neuropathic pain without eliciting any hallucinogenic behaviors [40].

This capacity for multi-target modulation equips classic psychedelics with substantial potential for intervening in highly heterogeneous and systemic neurological dysfunctions, although it simultaneously poses a significant challenge in dissecting their precise analgesic mechanisms. The clinical management of chronic pain has long been impeded by a fundamental therapeutic challenge: conventional analgesics predominantly focus on blocking ascending nociceptive transmission, yet they fundamentally fail to reverse the central sensitization and affective network rigidity perpetuated by persistent pain [3,4,14]. The profound neuroplastic alterations induced by psychedelics offer a novel avenue to breach this complex pathological impasse [28,41]. Building upon this foundation of multi-target network modulation, it is imperative to re-examine how psychedelics drive a fundamental paradigm shift in chronic pain treatment—transitioning from a conventional sensory blockade model to a strategy focused on the systemic reconstruction of maladaptive pathological networks.

## 3. Potential Therapeutic Efficacy of Psychedelics in Chronic Pain

A substantial body of preclinical and clinical evidence supports the vital analgesic potential of psychedelics [43,44,45,46]. In murine models, a single dose of psilocybin elicits a sustained antinociceptive effect against chronic neuropathic pain and inflammatory pain and significantly potentiates the antinociceptive efficacy of gabapentin [47,48]. Systemic administration of psilocybin, acting via 5-HT2A receptor activation, dose-dependently reverses mechanical and cold allodynia in models of chemotherapy-induced peripheral neuropathy (CIPN) and chronic inflammation [44]. Similarly, ayahuasca produces enduring analgesic effects in experimental neuropathy models [37]. However, a recent study shows that single-dose psilocybin, across multiple murine pain models, including spared nerve injury (SNI), complete Freund’s adjuvant (CFA) and acid-induced muscle pain (AIMP) models, has no evidence of immediate or persistent analgesia across diverse sensory, functional, and affective behavioral assays [49]. The only observed effect, a reduced sensitivity to cold, was attributed to psilocybin-induced profound hypothermia rather than true antinociception [49].

Overall, preclinical research on psychedelic analgesia remains at an early stage and is characterized by considerable methodological heterogeneity. The discrepancies between available findings may partly reflect differences in dosing regimens, routes of administration, pain models, and the timing of behavioral assessments relative to the acute psychoactive phase [47,48,49]. Furthermore, a major limitation of current preclinical designs is the over-reliance on stimulus-evoked reflexive assays (e.g., von Frey), which may be confounded by acute psychedelic-induced physiological changes, such as hypothermia and motor suppression. Future evaluations should complement non-reflexive, voluntary and affective-motivational behavioral assays—such as conditioned place preference (CPP), the mouse grimace scale (MGS) or evaluating the restoration of natural instinctual behaviors like burrowing and nest building—to reflect improvements in overall well-being and motivation [50,51].

In clinical settings, despite these preclinical discrepancies, the application of psychedelics has demonstrated favorable analgesic outcomes across diverse refractory conditions. At the epidemiological level, Cavarra et al. conducted an online survey disseminated through the Beckley Foundation website and social media, initially recruiting 976 respondents with current or previous chronic pain. Of these, 170 participants who completed the survey were ≥18 years of age, had prior psychedelic experience, provided pain-relief data, and met the predefined inclusion criteria for one of five chronic pain conditions (fibromyalgia, arthritis, migraine, tension-type headache, or sciatica) were included in the final analysis. The survey found that participants perceived psychedelics as providing greater analgesic efficacy than conventional medications for fibromyalgia, arthritis, migraine, and tension-type headache, whereas no significant advantage was observed for sciatica [52]. A recent clinical case report highlighted the therapeutic potential of psychedelics for severe chemotherapy-induced neuropathic pain in a 64-year-old male patient [53]. Upon receiving 3,4-methylenedioxymethamphetamine (MDMA), the patient, who received a repeated low dose (once every two days, 12.5–25 mg per dose, for four months), experienced significant improvement in neuropathic pain, with the analgesic efficacy persisting even after the cessation of repeated MDMA administration [53]. Expanding this clinical scope, a recent open-label pilot study explored the efficacy of psilocybin-assisted therapy in patients with post-treatment Lyme disease (PTLD)—a chronic sequela affecting 10–20% of Lyme disease survivors, characterized by intractable pain, fatigue, and mood disturbances [54]. The study enrolled 20 patients (11 females, 9 males; mean age 44 years) with a confirmed PTLD diagnosis who underwent a structured 8-week intervention comprising two psilocybin dosing sessions (15 mg at week 4, followed by 15 or 25 mg at week 6) coupled with psychological support. The clinical outcomes were promising: pain, fatigue, mood, and sleep quality exhibited significant and sustained amelioration across the 6-month period [54]. However, despite these enduring positive trends, the methodological quality of this study is inherently constrained by its open-label design and small sample size without a placebo control group. The conclusions remain tentative until validated by adequately powered, randomized, double-blind controlled trials. However, a recent randomized, double-blind, placebo-controlled, within-subject study evaluated the analgesic potential of non-hallucinogenic, low doses of LSD in 24 healthy volunteers (12 males, 12 females; mean age 22.7 years) [55]. Participants received single oral doses of 5, 10, and 20 µg of LSD and a placebo on separate days, followed by a cold pressor test (3 °C) to experimentally induce pain. The results demonstrated that the 20-µg dose of LSD significantly increased pain tolerance by approximately 20% and decreased subjective ratings of painfulness and unpleasantness at both 1.5 and 5 h post-administration [55]. Importantly, these sustained analgesic effects were achieved without inducing profound mind-altering psychedelic experiences or clinically relevant cardiovascular changes, revealing the threshold dose at which LSD exerts pain relief with minimal mental interference [55]. Furthermore, in the context of neuroimmune disorders, a case study reported on two multiple sclerosis (MS) patients (a 41-year-old male and a 44-year-old female) treated with a massive flood dose of ibogaine hydrochloride (up to 1200 mg) under 24 h cardiac monitoring, followed by a daily microdosing maintenance regimen (20 mg/day) [56]. The clinical and neuroimaging outcomes were striking: One patient demonstrated a 71% reduction in lesion volume and decreased apparent diffusion coefficient (ADC) values, suggesting active remyelination and reduced inflammation [56]. Both patients exhibited cortical and subcortical alterations, particularly in regions associated with pain and emotional processing, which correlated with significant clinical improvements in mobility, pain, and fatigue [56]. Moreover, recent evidence suggests that psilocybin holds novel therapeutic promise in alleviating phantom limb pain, paving the way for its integration with adaptive sensory technologies [57]. These observations suggest that psychedelics show great potential for pain management, and the precise biological mechanisms underlying psychedelic-mediated analgesia remain an active area of investigation.

## 4. Possible Mechanisms of Analgesia by Classic Psychedelics

Given the distinct chemical structures and polypharmacological profiles of psychedelics, characterized by their broad affinities for both serotonergic and non-serotonergic monoaminergic receptors, as well as their capacity to engage unique receptor heterodimerization cascades, their multidimensional analgesic potential typically extends across multiple functional domains of the nervous system [25,26]. To systematically analyze this complex, multi-target biological regulation, this section delineates the underlying mechanisms of psychedelic-induced pain relief through a structured hierarchy, tracing from peripheral to central integration and from microscopic neuronal plasticity changes to macroscopic functional connectivity of brain regions.

### 4.1. Serotonergic System

The transduction, central processing, and descending gating of nociceptive signals rely on highly orchestrated neurotransmitter networks [58,59,60]. Among these, serotonin (5-HT)—a ubiquitous monoamine transmitter distributed across the peripheral and central nervous systems (CNS)—emerges as a core modulator in diverse physiological processes and neuropsychiatric conditions, spanning depression, anxiety, schizophrenia, obesity, and pain [61,62]. Given the profound clinical comorbidity between chronic pain and affective disorders, the serotonergic system, serving as a critical bridge between sensory perception and affective processing, has increasingly become a frontier focus in pain medicine [63,64,65].

Anatomically, the somata of central serotonergic neurons are distinctively clustered within the brainstem raphe nuclei [62]. Their ascending fibers project diffusely to higher-order brain regions, including the hippocampus, midbrain, prefrontal cortex, parieto-occipital cortex, ACC, and thalamus [62,66]. Conversely, descending projections originating from the caudal raphe nuclei primarily innervate the cerebellum and spinal cord segments [62,66]. This extensive topographical distribution endows the serotonergic descending pathway with a highly bidirectional capacity—descending inhibition versus facilitation—for modulating spinal nociceptive transmission [67,68]. The ultimate functional output is strictly contingent upon the pathological phase of pain (acute or chronic), the spatial localization of the receptors, and the specific receptor subtypes engaged [67,68].

Based on their pharmacological and structural profiles, 5-HT receptors are classified into seven subfamilies (5-HT1 to 5-HT7), comprising at least 14 distinct subtypes encoded by independent genes [62]. While the pro-nociceptive role of peripheral 5-HT is universally acknowledged, the precise contributions of different receptor subtypes to nociceptive transmission at spinal and supraspinal levels remain a subject of considerable debate [69,70,71]. Current evidence indicates that spinal microcircuits are enriched with at least three 5-HT receptor families (5-HT1, 5-HT2, and 5-HT3) that exhibit differential affinities for 5-HT [62]. Furthermore, the recently characterized excitatory 5-HT7 receptor has garnered substantial attention due to its pivotal involvement in migraine pathophysiology, circadian rhythms, and thermoregulation [72].

Contemporary clinical paradigms dictate that the effective management of chronic pain necessitates concurrent therapeutic interventions targeting its negative affective dimensions. As previously discussed, classic psychedelics—acting as potent ligands for the serotonergic system, particularly the 5-HT2 receptor family—have recently demonstrated paradigm-shifting clinical potential in treating refractory affective disorders and chronic pain [18,30,34]. It is reasonable to hypothesize that this dual pharmacological advantage of psychedelics, combining robust analgesia with affective restoration, is rooted in the vast and exquisitely orchestrated “brainstem–cortex–spinal cord” multiscale regulatory network of the 5-HT system.

#### 4.1.1. Peripheral/Dorsal Root Ganglion Effects

**Figure 2:**
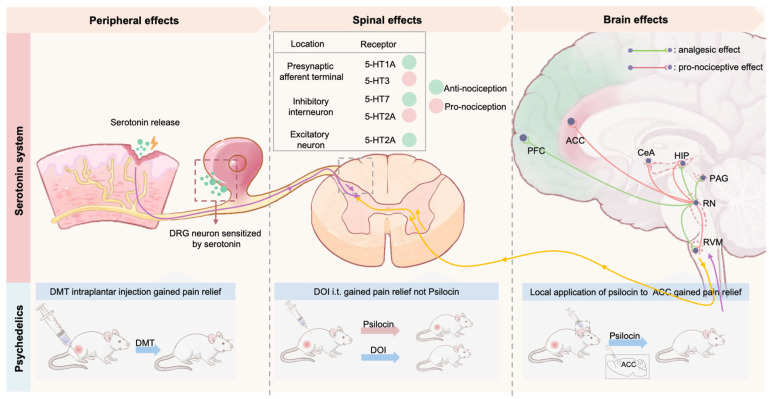
Modulatory effects of the serotonergic system and psychedelics on pain across different neural levels. **Peripheral effect:** In the peripheral system, tissue injury triggers the local release of 5-HT, which consequently sensitizes peripheral nociceptors. Paradoxically, intraplantar injection of the psychedelic DMT produces a significant analgesic effect. **Spinal effect:** At the spinal level, intrathecal injection of 5-HT generally exerts an overall analgesic effect [84]. However, this modulation is heterogeneous depending on the specific receptor subtype: activation of 5-HT1A (located on presynaptic afferent terminals [111]) and 5-HT7 (located on inhibitory interneurons [112]) receptors mediates antinociception, whereas 5-HT3 receptor (located on presynaptic afferent terminals [111]) activation drives pro-nociception, and 5-HT2A receptor activation demonstrates a bidirectional role and is located on both excitatory neurons and inhibitory interneurons [111]. Regarding psychedelic interventions, intrathecal administration of DOI induces analgesia, whereas psilocin injection yields no such effects. **Brain effect:** In the brain, the somata of serotonergic neurons are predominantly clustered in the raphe nuclei (RN), from which they project extensively: ascending to regions such as the prefrontal cortex (PFC), anterior cingulate cortex (ACC), central amygdala (CeA), and hippocampus (HIP), and descending to structures including the periaqueductal gray (PAG) and rostral ventromedial medulla (RVM). The arrows in the schematic indicate the approximate projection networks of serotonergic neurons (note: they do not represent precise anatomical pathways). Green arrows denote pathways where activation produces an analgesic effect (e.g., projections to the PFC and PAG [104,113,114,115]); red arrows indicate pathways where activation induces a pronociceptive effect (e.g., projections to the ACC and CeA [116,117]); projections to the RVM and HIP exhibit bidirectional modulatory functions, mediating both pronociceptive and antinociceptive outcomes [118,119,120]. Furthermore, local application of psilocin in the mouse ACC yields a notable analgesic effect. This figure was created and assembled by the authors using Adobe Illustrator 2023, Procreate 5.4.11 and Microsoft PowerPoint 16.89.1.

Extensive human and preclinical studies have established that peripherally administered serotonin (5-HT) acts predominantly as a pro-nociceptive mediator [73,74,75]. During peripheral tissue injury or inflammation, 5-HT released from platelets and mast cells accumulates at the site of damage, where it directly binds to the terminals of primary afferent sensory neurons and the somata of dorsal root ganglion (DRG) neurons, thereby activating pain-transmitting pathways (Figure 2) [76]. Furthermore, prolonged exposure to a pro-inflammatory microenvironment induces peripheral sensitization in sensory neurons, significantly lowering their activation thresholds for subsequent nociceptive stimuli (Figure 2) [58,77]. DRG neurons, particularly unmyelinated C-fibers and thinly myelinated Aδ-fibers, broadly express a myriad of receptor subtypes, including 5-HT1B, 5-HT1D, 5-HT2A, 5-HT2C, 5-HT3, and 5-HT7 [78]. Importantly, under pathological conditions such as inflammation or nerve injury models (e.g., spinal nerve ligation [SNL], chronic constriction injury [CCI]), the expression of 5-HT2A, 5-HT2B, 5-HT3A, and 5-HT7 receptors on DRG neurons is markedly upregulated [79,80]. For instance, in rat models of L5 SNL, subcutaneous or local administration of the 5-HT2A receptor antagonist ketanserin effectively alleviates mechanical hyperalgesia [81]. Mechanistic investigations have further revealed that nerve injury triggers the aberrant overexpression of neuropeptide Y (NPY) and calcitonin gene-related peptide (CGRP); ketanserin, by blocking peripheral 5-HT2A receptors, successfully counteracts this NPY and CGRP upregulation, thereby conferring anti-neuropathic pain effects [82].

Based on this pharmacological rationale, classic psychedelics, acting as potent 5-HT2A receptor agonists, should theoretically exacerbate pain in the periphery. However, recent experimental evidence has challenged this conventional pharmacological paradigm. Fonseca et al. (2026) discovered that local intraplantar injection of the classic psychedelic N,N-dimethyltryptamine (DMT) in rats not only failed to aggravate prostaglandin E2 (PGE2)-induced hyperalgesia but conversely produced a robust, dose-dependent peripheral analgesic effect (Figure 2) [43]. Notably, this effect exhibited high local specificity; even at the highest administered doses, DMT did not alter the nociceptive threshold in the contralateral, vehicle-injected paw. Pharmacological blockade experiments demonstrated that this peripheral analgesia was partially antagonized by selective 5-HT2A (ketanserin) and 5-HT3 (ondansetron) receptor antagonists [43]. More crucially, this analgesic effect was partially attenuated by cannabinoid type 1/type 2 (CB1/CB2) receptor antagonists and completely reversed by the non-selective opioid receptor antagonist naloxone [43]. These findings challenge the conventional view that activation of peripheral 5-HT2A receptors is uniformly pro-nociceptive. Instead, they raise the possibility that, under certain pathological conditions, classic psychedelics may engage peripheral endogenous analgesic pathways involving serotonergic, cannabinoid, and opioid signaling. However, the precise molecular and cellular mechanisms underlying these effects remain poorly understood, and the current evidence is limited to a small number of preclinical studies. Therefore, whether peripheral actions make a substantial contribution to the long-lasting analgesic effects observed in chronic pain remains to be determined.

While emerging evidence reveals a paradoxical peripheral analgesic role for classic psychedelics, the peripheral nervous system is currently regarded as a minor action site of psychedelic analgesia [45,83]. These localized effects alone are insufficient to account for the profound, long-lasting and systemic relief observed in complex chronic pain conditions. To fully comprehend the therapeutic mechanisms of these compounds, particularly their unique capacity to decouple nociceptive sensory input from entrenched affective and cognitive distress, scientific focus must shift upstream.

#### 4.1.2. Spinal Effects

The spinal dorsal horn, serving as the primary relay station for ascending nociceptive transmission, receives dense descending serotonergic projections from brainstem structures such as the rostral ventromedial medulla (RVM) [59,60]. The spinal cord widely expresses various 5-HT receptor subtypes [60]. During the pathological evolution of neuropathic pain, the modulatory role of the spinal 5-HT system exhibits a high degree of heterogeneity and bidirectionality; its ultimate net effect fundamentally depends on the specific receptor subtypes activated and the dynamic balance between descending inhibition and facilitation [67,68]. In preclinical pain models, contrary to the pro-nociceptive effects typically induced by peripheral application, direct intrathecal injection of 5-HT generally exerts significant overall antinociceptive effects [84].

Specifically, the activation of local spinal 5-HT1A, 5-HT7, and 5-HT2C receptors primarily mediates potent antinociception (Figure 2). Notably, the 5-HT1A receptor acts as a crucial hub for various non-opioid analgesics (such as curcumin, ferulic acid, and cannabidiol), as specific spinal blockade of this receptor reliably attenuates their anti-hyperalgesic effects [84,85,86]. Concurrently, 5-HT7 and 5-HT2C receptors induce analgesia by recruiting inhibitory GABAergic interneurons and triggering endogenous norepinephrine release, respectively [87,88,89,90]. Conversely, spinal 5-HT2A receptors demonstrate profound pharmacological heterogeneity. While their pathological activation typically drives feedforward pro-nociception via the mGluR/protein kinase C (PKC) pathways [91], specific targeted interventions (e.g., spinal cord stimulation) can paradoxically elicit 5-HT2A-mediated alleviation of hyperalgesia [92,93,94]. This diametrically opposed phenotype likely stems from the receptor’s heterogeneous expression across both inhibitory interneurons and excitatory projection neurons, with the ultimate functional output dictated by the predominant cell population recruited during varying interventions [60,68].

While these extensive investigations into spinal 5-HT receptor mechanisms provide a compelling theoretical framework for psychedelic-mediated analgesia, emerging evidence reveals a discrepancy between generalized 5-HT-based spinal modulation and the actual pharmacological behavior of classic psychedelics. In rat SNL models, an intrathecal injection of DOI can produce analgesia (Figure 2) [87]. However, a recent study also shows that although psilocybin exhibits a remarkably potent and enduring analgesic effect following intraperitoneal injection, direct targeted delivery of psilocin to the spinal level via intrathecal injection failed to alleviate mechanical allodynia in mice (Figure 2) [48]. This pharmacological divergence may partly reflect differences in chemical scaffold, receptor engagement, and signaling bias between DOI and psilocin, although the precise mechanisms remain speculative.

#### 4.1.3. Brain Effects

The brain is not merely the ultimate hub for processing the sensory-discriminative dimensions of nociceptive signals; it serves as the neuroanatomical core for encoding pain-associated emotions and orchestrating descending pain modulation [2,64,95]. In the supraspinal nervous system, 5-HT receptors exhibit a broad and highly specific spatial distribution, establishing the neurotransmitter foundation for the high clinical comorbidity between chronic pain and affective disorders [62,63,68]. Extensive basic research has demonstrated that multiple subtypes of the 5-HT receptor family play indispensable roles within the brain’s pain matrix and limbic system. For instance, 5-HT1A and 5-HT2A receptors display remarkable expression densities in the frontal cortex, ACC, insula, and amygdala, deeply engaging in the cognitive evaluation and affective processing of persistent pain [96,97]. Meanwhile, the 5-HT7 receptor, highly enriched in the hippocampus and hypothalamus, not only intervenes in emotional regulation and memory consolidation under pain conditions via the activation of the extracellular signal-regulated kinase (ERK) pathway [98,99,100], but its specific localization in the suprachiasmatic nucleus (SCN) also designates it as a critical molecular switch for central circadian rhythm regulation [72]. Furthermore, essential nodes of the descending pain control network—such as the RVM, ventrolateral periaqueductal gray (vlPAG), and raphe nuclei—are similarly enriched with diverse 5-HT receptors [59,60]. Somatodendritic 5-HT1A autoreceptors within the raphe nuclei potently modulate overall pain sensitivity, whereas excitatory synaptic transmission in the central nucleus of the amygdala (CeA) can trigger robust descending pro-nociceptive facilitation via brainstem projection pathways, strictly dependent on spinal 5-HT3 receptors [101,102].

As traditional peripheral or spinal-targeted analgesic strategies encounter clinical bottlenecks, the dual potential of classic psychedelics for analgesia and affective restoration in the central nervous system has garnered immense attention. Current mechanistic investigations have pinpointed the core target of psychedelic intervention for chronic pain to higher-order cortical networks, prominently the ACC [48]. Retrospective examination of 5-HT-mediated cortical analgesic mechanisms reveals that traditional monoreceptor pharmacological interventions often yield controversial conclusions. In nerve-injured rat models, microinjection of 5-HT2 family agonists into the ventrolateral orbital prefrontal cortex (vlOFC) produces significant anti-hyperalgesic effects, while knocking down 5-HT2A receptors in this region exacerbates mechanical hypersensitivity and spontaneous pain [103,104]. Paradoxically, Viscarra et al. found in a similar model that intracortical injection of a 5-HT2A antagonist (volinanserin) also effectively alleviates hyperalgesia by coupling with inhibitory potassium channels (KV7.x) [105]. This paradoxical pharmacological phenotype—where both agonism and antagonism confer analgesia—strikingly highlights the heterogeneity of cortical analgesic networks and suggests the unique mechanistic action of psychedelics. Under prolonged chronic pain stress, pyramidal neurons in cortical regions such as the ACC often lapse into a state of pathological, sustained hyperactivity [48,106]. Recent in vivo calcium imaging studies confirm that psilocin can specifically and rapidly suppress the aberrant high-frequency firing of layer 2/3 pyramidal neurons in the ACC, thereby achieving normalization of pain-related cortical hyperactivity [48].

Notably, psychedelic-induced neuromodulation here exhibits extreme pharmacological complexity: neither standalone 5-HT2A nor 5-HT1A agonists can fully mimic the analgesic phenotype of psilocin, and its effect on ACC pyramidal excitability is not unidirectional suppression [48]. Research reveals that local administration of psilocin into the ACC of healthy wild-type mice paradoxically increases neuronal calcium signals, whereas in spared nerve injury mice, it induces a marked suppression of calcium signals followed by profound analgesia (Figure 2) [48]. This diametrically opposed response suggests that psychedelic modulation of cortical microcircuits is characterized by a state-dependent homeostatic reset. Neuroanatomical evidence further bolsters this hypothesis: 5-HT2A receptors are not only densely enriched on the somata and apical dendrites of layer 5 excitatory pyramidal neurons in the PFC but also exhibit critical distribution among middle-layer GABAergic interneurons and deep-layer (layer 6b) non-pyramidal cells [107,108]. This heterogeneous hierarchical distribution endows the cortex with highly resilient, differential control capabilities over nociceptive signaling. However, the precise molecular and circuit-level mechanisms by which psychedelics accurately “sense” and integrate the prevailing baseline activity state of neural networks, subsequently translating this into targeted downstream signaling cascades to restore physiological homeostasis, remain a major unelucidated gap in the field.

Importantly, the local cortical homeostatic reset induced by psychedelics is frequently accompanied by systemic remodeling of large-scale, cross-neurotransmitter functional connectivity across the entire brain. Crucially, this targeted intervention within the serotonergic pathway triggers profound cross-transmitter cascades. For instance, a seminal human positron emission tomography (PET) study demonstrated that low-dose psilocybin (0.25 mg/kg), mediated by the activation of 5-HT1A/2A receptors, provokes a robust release of dopamine within the striatum [109]. At the macroscopic systems-imaging level, a dual-regression analysis further revealed that psilocybin (1–2 mg/kg) not only significantly enhances functional connectivity among the cerebral cortex, thalamus, and midbrain—a topographical pattern highly congruent with regions of elevated 5-HT2A gene expression—but also specifically attenuates connectivity within the dopaminergic ventral striatal pathway [110]. This capacity to transcend a single neurotransmitter system and precipitate global neural network reconfiguration provides a compelling macroscopic neuroimaging foundation for understanding how psychedelics systematically modulate nociceptive perception alongside dopaminergic reward and affective processing.

### 4.2. Anti-Inflammatory and Neuroimmune Modulatory Mechanisms

The pathological progression of chronic pain is driven not only by the structural remodeling of neural circuits but also by the persistent, aberrant activation of the immune system [121,122]. Within classical immunopathology, 5-HT has long been characterized as a central pro-inflammatory mediator. At sites of tissue injury or chronic inflammation, locally released 5-HT binds to 5-HT2A and other receptors on primary afferent neurons and immune cells (such as macrophages) [76,123]. This engagement promotes the secretion of pro-inflammatory cytokines, such as interleukin-6 (IL-6) and tumor necrosis factor-alpha (TNF-α), thereby directly driving peripheral sensitization and exacerbating hyperalgesia [124,125]. Consistent with these observations, peripheral 5-HT2A receptor activation has been shown to potentiate inflammatory nociception, whereas pharmacological blockade of 5-HT2A receptors can attenuate hyperalgesia in experimental inflammatory pain models [126,127].

Paradoxically, classic psychedelics exhibit a striking and counterintuitive pharmacological profile in the realm of immune regulation. Despite acting as potent 5-HT2A receptor agonists, psychedelics fail to elicit pro-inflammatory responses; instead, they demonstrate profound anti-inflammatory potential [128]. In in vitro models of rat aortic smooth muscle cells, the classic psychedelic (R)-DOI, at exceptionally low picomolar concentrations (IC50 = 10–20 pM), potently abrogates TNF-α-induced nuclear factor-κB (NF-κB) activation and its subsequent nuclear translocation [129]. Concurrently, it comprehensively downregulates the gene expression of intercellular adhesion molecule 1 (ICAM-1) and vascular cell adhesion molecule 1 (VCAM-1) and pro-inflammatory cytokines (IL-6, IL-1β) [129]. This phenotypic inversion from a pro-inflammatory to an anti-inflammatory state is currently attributed to biased agonism at the receptor level [130,131]. These divergent inflammatory outcomes may reflect ligand-dependent functional selectivity at 5-HT2A receptors. Psychedelic agonists such as (R)-DOI can produce potent 5-HT2A-dependent anti-inflammatory effects, whereas the precise signaling events that distinguish these responses from serotonin-associated pro-inflammatory actions remain incompletely defined [128,130,131].

Crucially, a spectrum of in vivo animal models, including those for asthma and cardiovascular inflammation, has corroborated that this robust anti-inflammatory efficacy is achieved at sub-behavioral doses, far below the threshold required to induce psychedelic-like behaviors such as head-twitch responses [128,132]. Furthermore, unlike conventional broad-spectrum immunosuppressants such as corticosteroids, psychedelics exhibit highly precise immunomodulatory properties [128,133,134]. They selectively and potently suppress hyperactive pathological inflammatory factors while sparing other immune parameters essential for basal defense. This targeted action largely preserves the integrity of the host immune response, thereby substantially mitigating the risk of opportunistic infections [133,134].

Integrating these unique neuroimmune modulatory properties into the therapeutic framework for chronic pain provides a critical missing piece in deciphering the analgesic mechanisms of psychedelics. Under chronic pain conditions, the aberrant activation of glial cells within the central nervous system (particularly in the spinal cord and higher-order cortical regions) and the concomitant massive release of cytokines, such as TNF-α and IL-1β, are widely recognized as the core engines driving and sustaining central sensitization [121,122]. This inflammatory milieu locks nociceptive networks into a state of persistent hyperexcitability. Recent studies have definitively shown that psilocybin not only reduces the concentrations of pro-inflammatory cytokines in in vitro cultures of lipopolysaccharide (LPS)-stimulated macrophages but also effectively downregulates cytokine levels in the murine brain following in vivo LPS exposure [135,136]. This may cultivate a permissive, anti-inflammatory milieu conducive to the repair of damaged synapses. Consequently, it is probable that the immune-modulating mechanisms of psychedelics operate synergistically with their capacity to induce structural synaptic plasticity (detailed in Section 4.3).

### 4.3. Structural Neuroplasticity of Classic Psychedelics

Human neuroimaging reveals that chronic pain induces macroscopic gray matter atrophy in higher-order regions like the mPFC and ACC [137,138,139]. This atrophy is reversible following efficacious behavioral or pharmacological interventions, indicating that it is driven by maladaptive neuroplastic remodeling rather than irreversible neurodegeneration [12,140]. Crucially, at the microstructural level, this pathological remodeling does not manifest as a homogeneous decline; rather, it exhibits pronounced regional and layer-specific heterogeneity. For instance, layer 5 pyramidal neurons in the PFC exhibit dendritic retraction and spine loss, whereas layer 2/3 neurons in both the ACC and PFC display increased dendritic arborization and elevated spine density [141,142]. These divergent structural alterations are mirrored by corresponding electrophysiological changes; for example, the excitability of ACC layer 2/3 neurons is robustly enhanced, whereas layer 5 neurons exhibit an opposing trend of reduced excitability [143]. Collectively, these findings suggest that chronic pain orchestrates a highly heterogeneous pathological transformation of neural circuits across spatial, structural, and functional dimensions.

**Figure 3:**
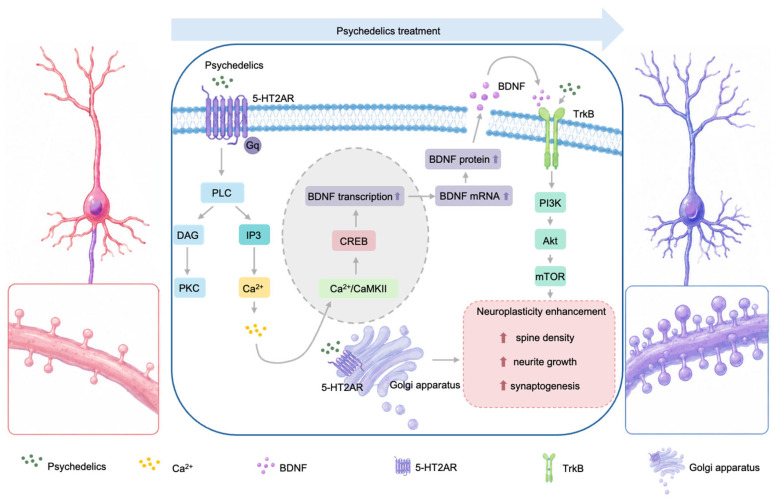
Mechanisms of psychedelic-induced structural neuroplasticity. The schematic illustrates that psychedelics promote neuroplasticity by: (1) activating cell-surface 5-HT2AR linked to Gq–PLC (phospholipase C) signaling, resulting in increased BDNF protein, which binds to TrkB and promotes enhanced neuroplasticity [38,146]; (2) directly binding to and activating the neurotrophic TrkB receptor [147]; and (3) traversing the plasma membrane to uniquely activate intracellular 5-HT2AR populations localized on the Golgi apparatus [144]. This figure was created and assembled by the authors using Procreate 5.4.11 and Microsoft PowerPoint 16.89.1.

Classic psychedelics, acting as potent inducers of neuroplasticity (psychoplastogens), appear capable of reversing these microstructural pathologies. Highly lipophilic psychedelics, such as DMT and its derivatives, readily traverse the cell membrane not only to activate cell-surface 5-HT2A receptors but also specifically bind to and activate intracellular 5-HT2A receptors localized on endomembrane systems (in the Golgi apparatus, Figure 3) [144]. Furthermore, they can directly bind to and activate TrkB with exceptionally high affinity (Figure 3) [38]. This dual-activation mechanism triggers intracellular signaling cascades, prominently including mechanistic target of rapamycin (mTOR), which subsequently drives massive synaptogenesis and neuritogenesis in the mPFC within hours to days post-administration (Figure 3) [28,145]. A single administration of psilocybin rapidly promotes dendritic spine formation and increases spine density in frontal cortical pyramidal neurons within 24 h, with structural remodeling persisting for up to one month [145]. This profound structural remodeling is accompanied by enhanced excitatory neurotransmission and effectively ameliorates stress-induced behavioral deficits [145]. This multidimensional mechanism—spanning the activation of both intra- and extracellular receptors alongside direct neurotrophic receptor engagement—may elegantly explain why psychedelics can rapidly drive synaptic remodeling and alleviate clinical symptoms within mere hours, whereas conventional pharmacotherapies typically require weeks to months to achieve efficacy.

However, conceptualizing psychedelics exclusively as unidirectional “promoters of growth” introduces a theoretical paradox when considered alongside the layer-specific pathology of chronic pain. If chronic pain already induces dendritic hypertrophy and increased spine density in layer 2/3 neurons, would the pan-synaptogenic effects of psychoplastogens not paradoxically exacerbate this local maladaptation? This theoretical gap underscores a critical frontier for future research. It is imperative that forthcoming mechanistic studies pivot from bulk-tissue dendritic spine quantification to precise, circuit- and layer-specific morphological analyses.

### 4.4. Breaking Chronic Pain-Induced Brain Network Rigidity and Resetting the Default Mode Network (DMN)

Neuroplastic changes at the microscopic scale inevitably integrate into functional state transitions at the brain-wide systemic level. At the macroscopic network scale, accumulating neuroimaging and theoretical evidence suggests that the hub of psychedelic-mediated analgesia likely converges on the brain’s DMN [5,148,149,150]. The DMN was described not as a single monolith but as a composite of multiple intertwined, parallel subnetworks [151,152]. These subnetworks serve as the foundation for individuals to maintain internal self-narratives, retrieve past experiences, and generate future expectations or prior beliefs [153,154].

In the state of chronic pain, this network not only suffers from disrupted internal functional connectivity but also loses its dynamic equilibrium in interacting with other brain regions [5,155,156]. The DMN’s abnormally enhanced internal connectivity continuously amplifies negative expectations and pain catastrophizing [5,153], fundamentally altering the weighting of bottom-up nociceptive signals [11]. Furthermore, several functional magnetic resonance imaging (fMRI) studies on patients with chronic low back pain have demonstrated that stronger connectivity between the DMN and the insula (a region known to be involved in pain processing [95]), coupled with weaker connectivity to the anterior cingulate cortex (a region involved in pain inhibition [157]), correlates with higher clinical pain severity [149].

**Figure 4:**
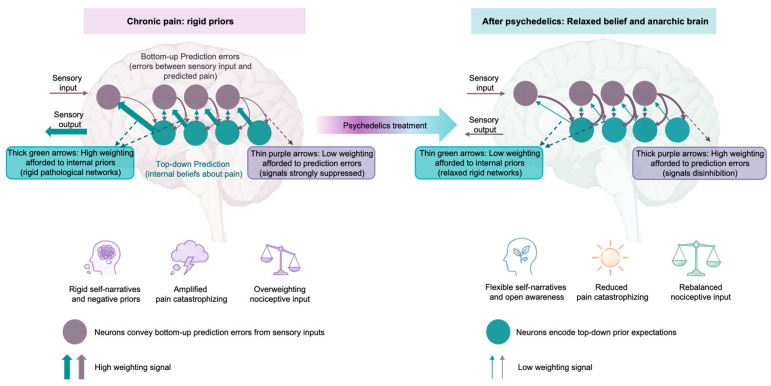
Psychedelic-induced modulation of precision weighting in hierarchical predictive coding. This schematic illustrates how psychedelics alter the balance between sensory input and internal expectations, based on the REBUS (Relaxed Beliefs Under Psychedelics) model and the anarchic brain. (Left) Chronic Pain State: Within the predictive coding hierarchy, purple circles represent neuronal populations (e.g., superficial pyramidal cells) that convey bottom-up prediction errors from sensory inputs. Green circles represent deep-layer networks (e.g., deep pyramidal cells) that encode top-down prior expectations. Prior to treatment, the thick green arrows indicate the high “precision” (or weighting) afforded to internal prior beliefs, resulting in dominant top-down predictions. Conversely, the thin purple arrows indicate that bottom-up sensory inputs (prediction errors) are strongly suppressed. This state is characteristic of rigid pathological priors, such as entrenched negative expectations in chronic pain or depression. (Right) Psychedelic Treatment State: Psychedelic treatment functionally decreases the precision of high-level prior expectations, represented by the thinning of the green arrows, which means a “relaxation” of top-down control. This disinhibition sensitizes the brain to actual ascending sensory input signals. Consequently, the thick purple arrows illustrate that bottom-up prediction errors are granted higher precision, and this mechanism breaks rigid pathological networks, freeing the brain to reintegrate authentic sensory inputs. This figure is adapted from Robin Carhart-Harris (2019) [160]. This figure was created and assembled by the authors using Procreate 5.4.11 and Microsoft PowerPoint 16.89.1.

Confronted with this neural rigidity, classic psychedelics exhibit a unique capacity to reset the network (Figure 4) [158,159]. Through 5-HT2A receptor agonism, they trigger the acute disintegration of intra-DMN connectivity while increasing connectivity with globally segregated networks, substantially elevating global brain entropy [148,160]. This disintegration disrupts the DMN’s excessive control over internal pain narratives, relaxing rigid pathological priors [148,161]. Upon homeostatic network resetting after acute effects subside, the brain is freed to reintegrate authentic bottom-up sensory inputs, which may ultimately break the chronic pain cycle [158]. However, recent findings caution that psychedelic-induced fMRI signal changes do not solely represent pure neuronal reorganization. Rather, they reflect a mixed effect involving the disruption of inherent neurovascular coupling mechanisms, highlighting the critical need to account for vascular effects when interpreting blood-oxygen-level-dependent (BOLD)-fMRI brain function measurements [162].

### 4.5. Remodeling Biological Rhythms in Cluster Headache and Migraine

Cluster headache (CH) and migraine, as highly debilitating primary headache disorders, are characterized most distinctively by their deeply entrenched periodicity and temporal rhythmicity [163,164]. Up to 70.5% of CH patients and 50.1% of migraine patients experience attacks at nearly identical times each day [164]. Current mainstream consensus posits that the paroxysmal eruption of these intractable headaches necessitates the coordinated engagement of the trigeminovascular system, the parasympathetic nervous system, and the hypothalamus [165,166]. The trigeminovascular system, which transmits craniofacial nociceptive signals, is tightly gated by the hypothalamic circadian rhythm apparatus [167]. Concurrently, recent investigations have further revealed that the peripheral trigeminal ganglion (TG) itself possesses a robust, autonomous circadian oscillation, within which the 5-HT2A receptor has been identified as a direct clock-controlled gene [168].

Confronted with these headache disorders driven by chronobiological dysregulation, classic psychedelics have demonstrated profound therapeutic and prophylactic potential across multiple clinical studies for both CH and migraine. In a large-scale British cohort study (N = 11,419), a cross-sectional epidemiological analysis employing multiple logistic regression revealed that individuals with lifetime use of classic psychedelics exhibited a significant 25% lower odds of suffering from frequent severe headaches [169]. This macroscopic population-level evidence has been further complemented by real-world follow-ups of non-clinical cohorts: long-term observation of individuals adhering to sub-behavioral, regular microdosing regimens of psilocybin or LSD indicates that this paradigm—far below the hallucinogenic threshold—not only effectively blocks the periodic recurrence of acute headache attacks but also concurrently improves accompanying negative affective comorbidities [170]. Furthermore, Schindler and colleagues at Yale University have recently spearheaded a series of milestone randomized controlled trials (RCTs). In a double-blind, crossover trial for episodic migraine, a single low oral dose of psilocybin (0.143 mg/kg) was demonstrated to significantly and persistently reduce weekly migraine days and substantially attenuate the sensory intensity of attacks over the subsequent two weeks [171]. To optimize this clinical dosing paradigm, Schindler et al., in their exploratory 2025 RCT, horizontally evaluated the prophylactic efficacy of a single dose versus a repeated, pulsed regimen, confirming that a short-term pulsed administration of psilocybin achieved highly comparable and enduring long-term analgesic and preventive outcomes [172]. This spectrum of clinical data reveals that low-dose administration of classic psychedelics exerts long-lasting prophylactic efficacy without eliciting confounding hallucinogenic effects, suggesting that at least some clinical benefits may be partly dissociable from the subjective psychedelic experience itself and providing a robust clinical-pharmacological rationale for the development of sub-behavioral or non-hallucinogenic derivatives.

Recently, a foundational study on the acute anti-headache agent ergotamine has further elucidated the indispensable role of the serotonergic system in modulating rhythmic neural networks [173]. Ergotamine, a peripheral 5-HT2A receptor agonist that is restricted from crossing the blood–brain barrier (BBB), acts directly upon the trigeminovascular system [173]. Real-time bioluminescence monitoring and quantitative polymerase chain reaction (qPCR) analyses verified that ergotamine treatment significantly amplifies the circadian amplitude of *Per2::LucSV* reporter mouse fibroblasts as well as isolated trigeminal ganglion explants without disrupting period length [173]; concurrently, the transcriptional amplitudes of core clock genes (including *Clock*, *Bmal1*, *Period3*, *Cryptochrome2*, *Rev-erbα*, and *Rev-erbβ*) were comprehensively enhanced. Pharmacological antagonism experiments further demonstrated that this ergotamine-mediated amplification of clock amplitude was blunted by GR127935 (a 5-HT1B/1D antagonist), BRL1557 (a 5-HT1D antagonist), asenapine (a multi-target 5-HT1A/1B/2A/2B/2C, α1A, and D1–D4 antagonist), and SB242084 (a 5-HT2C antagonist), confirming that this chronobiological modulation is strictly mediated by the activation of the serotonin receptor system [173]. Finally, in a nitroglycerin (NTG)-induced chronic headache mouse model, ergotamine exhibited a profound chronotherapeutic effect, significantly elevating the mechanical hindpaw withdrawal threshold when administered during the daytime (Zeitgeber time 4 [ZT4]), whereas it yielded no therapeutic efficacy at night (ZT16) [173].

This finding confirms that targeting the clock amplitude of the peripheral trigeminal ganglion via serotonin receptors precipitates a time-dependent, potent analgesic effect. Based on the foundational evidence that the peripheral trigeminal ganglion harbors an intrinsic clock and that the 5-HT2A receptor is a clock-controlled gene, it is highly probable that classic psychedelics similarly exert long-lasting prophylactic effects against primary headaches by modulating biological rhythms. Compared with the limited CNS exposure reported for ergotamine, centrally active classic psychedelics or their active metabolites can access the brain and engage cerebral 5-HT2A receptors [144,174]. This implies that in the peripheral nervous system, classic psychedelics may mimic the pathway of ergotamine, activating 5-HT receptors within the trigeminal ganglion to regulate peripheral clock amplitudes. Concurrently, in the central nervous system, they may directly modulate the hypothalamus. Recent clinical neuroimaging research has provided direct objective support for this central hypothesis: following psilocybin treatment in patients with chronic cluster headache (CCH), the magnitude of functional connectivity (FC) enhancement between the hypothalamus and the diencephalon correlated significantly and negatively with headache attack frequency—meaning that the stronger the FC between the hypothalamus and the diencephalon, the fewer the headache episodes [175]. This neuroimaging evidence suggests that psychedelics may influence the hypothalamic circadian rhythm and strengthen its descending modulatory signals to the diencephalon or other brain regions, thereby suppressing aberrant trigeminal discharges and ultimately achieving therapeutic effects for primary headache disorders from a chronobiological dimension.

The further development of animal models of CH capable of replicating human rhythmic and autonomic symptoms represents a vital tool for validating these biological cellular mechanisms.

## 5. Efficacy Decoupling and the Blinding Dilemma: Core Challenges in the Clinical Translation of Psychedelics

A current mechanistic and translational debate in the field centers on whether the intense, altered state of consciousness (the hallucinogenic experience) is a prerequisite for therapeutic efficacy or merely a byproduct of 5-HT2A receptor activation [176,177]. On one hand, numerous clinical psychiatry trials and preliminary studies indicate that the intensity of ego dissolution experienced by patients during dosing is significantly and positively correlated with subsequent clinical benefits, including reduced pain severity [178,179]. On the other hand, recent breakthroughs in non-hallucinogenic neuroplasticogens challenge this paradigm [180,181,182]. A new generation of molecules, exemplified by Tabernanthalog (TBG, an ibogaine analog), demonstrates that in mouse models, even when the hallucinogenic liability is stripped away, significant enhancement of cortical plasticity and effective alleviation of neuropathic pain are still observed [181]. Similarly, clinical observations in CH indicate that the core mechanism by which psilocybin reduces attack frequency does not depend on the subjective hallucinogenic experience and that non-hallucinogenic psychedelics remain therapeutically effective for this condition [182]. The recently proposed concept of biased agonism further supports this view. The early Gq efficacy threshold hypothesis posited that hyperactivation of the Gq–PLC signaling pathway was central to triggering hallucinogenic experiences [183]; however, this view is currently being challenged by modern structural pharmacology. A recent study suggests that atypical Gi signaling mediated by the 5-HT2A receptor constitutes the true fundamental driver of hallucinogenic effects, whereas Gq signaling primarily mediates the therapeutic benefits [184]. By designing agonists with extreme Gq bias that are completely devoid of Gi activity (such as DIO-NBOMe), potent antidepressant effects and plasticity restoration can be achieved without hallucinogenic properties [184].

Given the significant contradiction between clinical results showing that hallucinogenic effects are associated with efficacy and basic research showing that these effects can be uncoupled at the molecular level, whether the hallucinogenic experience is a necessary component of therapeutic efficacy cannot be generalized. As noted previously, the experience of suffering is not solely a physiological nociceptive input; it arises from both physiological and psychological factors, encompassing an individual’s beliefs and expectations [11,185]. When patients undergo subjective experiences induced by psychedelics in clinical trials, these experiences may mechanistically diverge into two intertwined pathways. First, the marked acute subjective effects of classic psychedelics can substantially compromise blinding, making treatment allocation easier for participants and raters to infer and potentially interact with expectancy effects [5,186,187]. Correct identification of treatment allocation may alter treatment expectations, although the magnitude of this contribution to observed therapeutic efficacy remains uncertain. Second, this subjective experience may not merely be a placebo effect but rather a core therapeutic driver with independent value. The ego dissolution brought about by psychedelics may liberate a brain entrenched in catastrophizing, psychologically dismantling the patient’s fear-avoidance and rigidity regarding pain.

Whether reliant on the placebo effect or the intrinsic subjective experience of the psychedelic, these mechanisms, dependent on complex human cognition, are currently difficult to authentically model and evaluate in rodents. A major obstacle in establishing psychedelics as evidence-based analgesic therapies lies in the challenges of maintaining blinding. Because their acute psychoactive effects are difficult to mask, distinguishing genuine pharmacological efficacy from placebo or expectation effects becomes highly problematic. A recent randomized controlled trial on migraine prevention similarly exposed this issue: Although a psilocybin pulse regimen reduced migraine frequency by approximately 50%, the control group receiving diphenhydramine (as an active placebo) exhibited a statistically identical reduction [172]. This indicates that non-pharmacological contextual factors and patient expectations contribute substantially to the observed clinical differences. Consequently, how to achieve true blinding or select an appropriate chemical as a placebo remains critically important. Furthermore, future validation in human subjects through experience-blocking paradigms—such as administering classic psychedelics to subjects under general anesthesia and verifying whether analgesic effects are retained—could be instrumental. Through such validation strategies, we may genuinely achieve a physical uncoupling of underlying pharmacological actions from higher-order psychological experiences in human subjects, thereby unraveling the potential mechanisms of psychedelic therapy in chronic pain.

## 6. Conclusions and Future Insights

At present, the clinical management of chronic pain remains trapped in a formidable bottleneck. Because chronic pain is not merely a prolongation of peripheral tissue injury but is accompanied by profound affective disorders (e.g., depression, anxiety) and the pathological remodeling of central neural networks [2,10,188,189,190], conventional analgesics, including opioids and gabapentinoids, remain limited by incomplete efficacy and, for some agents, risks associated with long-term use, including tolerance, dependence, and misuse [191,192,193,194]. Against this backdrop, classic psychedelics have emerged as highly compelling candidates due to their unique pharmacological profiles, arguably representing a prototype for a “perfect” antinociceptive pharmacotherapy: They possess a rapid onset of action, elicit remarkably enduring therapeutic effects following a single dose or a short, pulsed regimen, lack addictive liability, and uniquely combine the alleviation of physical pain with the healing of negative affect [17,18]. This therapeutic potential endows them with unprecedented clinical promise in the management of primary headaches, neuropathic pain, and refractory pain associated with terminal illnesses.

As synthesized in this review, the mechanisms by which psychedelics exert their antinociceptive effects are highly complex; unraveling these mechanisms represents both the crux of future clinical translation and a prime target for basic preclinical investigation. At the microscopic level, acting as multi-target ligands (particularly at 5-HT receptors), psychedelics not only exert immunomodulatory and anti-inflammatory effects in the periphery but also rapidly drive structural and functional neuroplasticity within the central nervous system [38,128]. At the macroscopic level, psychedelics possess the unique capacity to disintegrate the brain network rigidity induced by chronic pain—particularly within the DMN [148]. Furthermore, psychedelics may play a pivotal role in modulating biological rhythms, which still need further exploration. These cross-scale network remodeling mechanisms may contribute to the sustained antinociceptive effects reported in some preclinical and clinical studies.

Given this immense potential, several critical questions must be addressed in ongoing and future preclinical and clinical studies: First, regarding routes of administration and spatial targeting, future research must more comprehensively compare the differential effects of systemic administration (e.g., intravenous or intraperitoneal injection) with central or localized delivery (e.g., intrathecal or intracerebral microinjection) across diverse neuropathic and inflammatory pain models in order to precisely delineate whether their primary analgesic mechanisms reside at the spinal level or within higher cortical/rhythm-generating centers. Second, sex, as an indispensable biological variable, bears core relevance to both chronic pain pathophysiology and responses to psychedelic treatments. Recent preclinical evidence indicates that 5-HT2A-associated cortical plasticity can exhibit sex-dependent patterns; therefore, future studies should systematically include both sexes and directly assess sex-by-treatment interactions [195]. Third, to address the most formidable hurdle in current clinical translation—the unblinding and expectancy effects driven by acute psychoactive properties—future research should strive to achieve efficacy decoupling between the underlying pharmacological actions and higher-order subjective experiences. Finally, beyond their potential to act as potent standalone antinociceptive agents, the capacity of psychedelics to modulate synaptic plasticity and network rhythms may also render them exceptional therapeutic “primers”. By preemptively optimizing the microenvironment of the nociceptive system, they could synergistically amplify the efficacy of co-administered analgesics [47].

In summary, classic psychedelics and their derivatives represent a transformative alternative or adjunct for the treatment of chronic pain. With the continued elucidation of their cross-scale analgesic mechanisms and biased signaling pathways, this once-stigmatized class of compounds is poised to deliver incalculable clinical value in breaking the intractable deadlock of chronic suffering in the future.

## Data Availability

No new datasets were generated in this study. All data and information discussed in this review are available in the cited publications and publicly accessible sources. During manuscript preparation, the authors used Gemini solely to assist with language editing, grammar correction, and improvement of readability. All AI-assisted content was critically reviewed and revised by the authors, who take full responsibility for the accuracy, integrity, and final content of the manuscript.
